# A targeted approach for multiplex detection of respiratory viruses in cases with severe acute respiratory infections by nanopore sequencing

**DOI:** 10.1371/journal.pone.0324601

**Published:** 2025-06-25

**Authors:** Arghavan Zebardast, Kaveh Sadeghi, Ahmad Nejati, Sevrin Zadheidar, Mohammad Hossein Najmi, Adel Abedi, Vahid Salimi, Mehdi Shabani, Jila Yavarian, Nazanin Zahra Shafiei Jandaghi, Talat Mokhtari Azad

**Affiliations:** 1 Virology Department, School of Public Health, Tehran University of Medical Sciences, Tehran, Iran; 2 Faculty of Biological Sciences, Department of Bioinformatics, Tarbiat Modares University, Tehran, Iran; 3 Mathematics Department, Shahid Beheshti University, Tehran, Iran; 4 Research Center for Antibiotic Stewardship and Antimicrobial Resistance, Tehran University of Medical Sciences, Tehran, Iran; Cairo University Faculty of Veterinary Medicine, EGYPT

## Abstract

Severe acute respiratory infection (SARI) remains one of the leading causes of morbidity and mortality worldwide. Multiple viruses can cause this infection. Sequencing technologies hold great promise for detecting viral pathogens. This proof-of-concept study aimed to develop and validate a new method for multiplex detection and typing of SARI-related viruses (SARS-CoV-2, Influenza A (H1N1, H3N2), Influenza B, human respiratory syncytial virus, human adenoviruses, human enteroviruses, and human parainfluenza viruses) using a nanopore next-generation sequencing method. Following genome extraction from oropharyngeal swab samples and conventional RT-PCR assays, the libraries were barcoded and sequenced by the MinION device. The sensitivity and specificity were assessed using various serial dilutions of samples and different primer pools, respectively. Data analysis was carried out using bioinformatic tools. Finally, the protocol was validated with known positive samples. All participants provided written informed consent. During 12 hours of MinION sequencing, 711,000 reads passed the quality filters (Q-score>7). Eleven out of 12 target genes were successfully identified in clinical samples, with more than 90% coverage for most viruses. All viruses were detected by a Q30 value of more than 1%. The detection limit was measured for SARS-CoV-2, Influenza A (H1N1, H3N2), Influenza B, and human respiratory syncytial virus. The method showed 99.9% specificity in detection and was validated by 20 clinical samples. This study developed and validated a novel multiplex detection approach of Oxford Nanopore Technologies that allowed the identification of SARI-related respiratory viruses in a clinical laboratory setting.

## Introduction

Severe acute respiratory infection (SARI) is a significant cause of morbidity and mortality worldwide, with 5.3 thousand deaths in 2020, in America [1,2]. The World Health Organization (WHO) defines SARI as an acute respiratory infection with an onset within the past 10 days, requiring hospitalization, and characterized by the presence of a cough and a fever of more than 38°C [3]. Viral infections account for 30–70 percent of SARI cases [4]. Many respiratory viruses cause similar symptoms that are not clinically distinguishable, like influenza viruses A and B (Flu A/B), human coronaviruses (HCoVs), human respiratory syncytial viruses (hRSV), human enteroviruses (HEVs), human adenoviruses (HAdVs), and human parainfluenza viruses 1-4 (HPIVs) [4,5].

Early diagnosis of the viral etiology of respiratory infections might prompt appropriate clinical management initiation [6]. Various techniques were used for diagnosing respiratory viruses, including immunofluorescence assays, polymerase chain reactions (PCRs), and singleplex or multiplex real-time reverse transcription PCR (multiplex RT-qPCR). Rapid multiplex virus identification is of great epidemiological and therapeutic interest [7]. Currently, the most widely used test for multiplex identification of respiratory viruses is multiplex multiplex RT-qPCR [8]. It should be noted that, during recent years, next-generation sequencing (NGS) technologies have found different applications in virology, such as metagenomics evaluation, whole-genome sequencing for surveillance, tracking the transmission of infectious agents, and finally, yet importantly, multiplex pathogen identification [9].

Third-generation sequencing (TGS) technologies, including Oxford Nanopore Technologies (ONT) and single-molecule real-time (SMRT) sequencing, are two long-read sequencing techniques frequently utilized in laboratories [10]. A palm-sized portable sequencer called MinION (Oxford Nanopore Technologies, Oxford, United Kingdom) provides real-time sequencing, which is more affordable and smaller than traditional sequencing devices [9]. MinION is especially useful where other platforms like Illumina, Ion Torrent, and PacBio are challenging [11]. Nanopore-targeted sequencing (NTS) is the fastest sequencing-based technology for multiplex respiratory virus identification [8].

It is noteworthy that clinical samples might be damaged before arriving at the lab, making virus detection challenging. In NTS, to enhance the rate of virus detection in clinical samples with low virus loads, the viral targeted genomes are amplified by conventional RT-PCR before sequencing.

Due to the co-circulation of respiratory viruses, the significant morbidity and mortality burden, the emergence of the severe acute respiratory syndrome coronavirus-2 (SARS-CoV-2), and a probable future pandemic, precise monitoring of SARI etiological agents is essential [6,12,13]. NTS could reduce the cost and the time required for virus detection during an outbreak, speeding up the response and assisting disease control [14]. This study developed and validated a new nanopore NGS approach for the multiplex detection of SARI-related viruses.

## Materials and methods

### Ethics approval and consent to participate

The research ethics committees of Tehran University of Medical Sciences, School of Public Health, and Allied Medical Sciences have approved the study with ID: IR.TUMS.SPH.REC.1401.192. For this investigation, OPS samples were provided via the Ministry of Health and Education and did not have direct patient interaction. Every procedure was carried out in compliance with the applicable institutional policies and standards.

### Sample collection

This is a proof-of-concept study. The oropharyngeal swab (OPS) samples from SARI cases were referred to the National Influenza Center of Iran. All participants provided written informed consent, which is documented in this center. Samples that were positive for respiratory viruses, including SARS-CoV-2, Flu A/(H1N1, H3N2), Flu B, hRSV, HAdVs, HEVs, and HPIV1–4, were selected to establish and evaluate the multiplex NTS for the detection of the mentioned viruses. These samples were collected between 10 January 2023 and 14 February 2024. After data collection, the authors had access to any information that may be used to identify specific individuals.

### Viral genome extraction

The viral genomes from OPS samples were extracted using a High Pure Viral Nucleic Acid kit (Roche Diagnostics, Germany), following the manufacturer’s instructions. The quantity of the extracted genomes was assessed spectrophotometrically using a Nanodrop 1000 (Thermofisher, United States). Extractions were divided into multiple aliquots and stored at −70 °C until molecular testing performance.

### Quantification of pathogens by real-time PCR

Every PCR and sequencing run contained negative control samples to ensure the accuracy of our findings and rule out any possible contamination. To determine the Ct value of each sample in singleplex reactions, rRT-PCR assays were performed using Invitrogen SuperScript III Platinum One-Step Quantitative RT-PCR (Thermo Fisher Scientific, USA) with specific primers and probes. The primer sequences, reaction mixture, and cycle condition were based on previous studies for each virus [15–20].

### Primer design for conventional RT-PCR

Conserved regions of the mentioned viruses’ target genes were chosen as candidate regions for detection, while the variable regions were selected for typing and variant identification. The primers of this study could simultaneously detect and type the specific viruses. For SARS-CoV-2 detection, some factors led to targeting both the spike (S) and nucleocapsid (N) genes. Targeting two genes improves the chance of finding the virus, mainly when the viral load is low, and a single gene target might not amplify well. This method lowers the possibility of false negatives while increasing test robustness. Moreover, providing the sequences of more than 1000 nucleotides of two genes (S and N) increases the specificity of this assay [21]. Another was mutations, which are common in the S gene, especially in emerging variants [22]. The highly conserved N gene is included to guarantee accurate detection of various SARS-CoV-2 strains [23]. This dual-target strategy helps mitigate the risk of missing new variants and the impact of sequence variations on the assay sensitivity [24]. To benefit from a MinION device’s long-read sequencing technology, amplicon sizes for all targets were set between 668 and 1298 base pairs (bp) [25]. For the primer design, the FASTA format of the reference target sequences was first collected from GenBank and GISAID databases. Then, the BioEdit Sequence Alignment Editor, Version 7.2.5 application, was used. Multiple sequence alignments (MSA) were performed using the ClustalW algorithm. Subsequently, the sequences were aligned, and proper regions for each target gene were selected, and multiplex PCR primers were designed. Analysis of physiochemical properties of all primers was assessed using the OligoAnalyser tool (https://www.idtdna.com). A nucleotide BLAST (Basic Local Alignment Search Tool) and primer designing tool analysis were utilized to verify the compatibility and specificity of designed primers (https://blast.ncbi.nlm.nih.gov/Blast.cgi & https://www.ncbi.nlm.nih.gov/tools/primer-blast/) (Table 1).

**Table 1:** The primer sequences of targeted genes.

Virus	Target gene	Accession number of the reference gene	Primer sequence (5’ → 3’)	Amplicon size (bp)
Flu A (H1N1)	HA	EPI_ISL_9142379 (Victoria)	F: AATGGAACGTGTTACCCAGGAG R: CAAATGTCCAGGAAACCATCATCAAC	998
Flu A (H3N2)	HA	EPI_ISL_12109641 (Darwin)	F: TGCCTCCCTTAGGTCACTAGTTG R: AACAAGAAGCTCMGCGTTGTATGAC	973
Flu B	HA	EPI_ISL_165887	F: CAAACTCACCTCATGTGGTCAAAAC R: CCTGCAATCATTCCTTCCCATCC	1069
SARS-CoV-2	N	OK083551.1	F: CTCAGATTCAACTGGCAGTAACCA R: GGAGAAATCATCCAAATCTGCAGC	1144
	S	NC_045512.2	F: ACTTTAGAGTCCAACCAACAGAATCT R: TGACTAGCTACACTACGTGCCC	1120
HPIV-1	HN	NC_003461.1	F: ACAGGAAYTGGCYCAGATATGCG R: TGCGTAYAGTGTGGTTGTAGC	1102
HPIV-2	HN	NC_003443.1	F: ACGCCTAAATATGGACCTCTCCT R: GGCTCCAGCAAATCGATAGTTG	1053
HPIV-3	HN	NC_001796.2	F: CAGGAGTGAATACAAGGCTTC R: GACATTCATTGTTTCCTGGTCTTG	1113
HPIV-4	HN	KX912854.1	F: CTGRGTCACAAGCCTCTACATGA R: GGATAGGGCCACCAGCTGGATC	902
HEVs	5´NCR	NC_038308.1	Fw: GCCCCTGAATGCGGCTAA Rev: CATCNGGNARYTTCCAVYACCA	668
HAdVs	Penton	DQ099432.4	Fw: CTAYCAGAAYGACCACAGCAACTT Rev: ATCTGGTTCTCRGGRAAGCGRTT	1298
hRSV	F	EPI_ISL_2991498	Fw: GCACCTAGAAGGGGAAGTGAAC Rev: GACTGGTGTGCTTCTGGCCTTG	1197

The variation in amplicon size was carefully considered during primer design so as not to negatively affect sequencing performance. Abbreviations: HA: hemagglutinin, N: nucleocapsid, S: spike, HN: hemagglutinin-neuraminidase, NCR: noncoding region, F: fusion.

### Conventional RT-PCR

Conventional RT-PCR assays were designed in three steps. In step one, conventional singleplex RT-PCRs were carried out using a one-step RT-PCR (Biotechrabbit, Germany) kit on the ProFlex thermal cycler (Thermo Fischer Scientific, USA). The reaction mixture and thermal condition for each assay are provided in Tables 2 and3. Sanger sequencing was conducted on each PCR product to confirm the target identification and the test specificity.

**Table 2:** The singleplex conventional RT-PCR reaction mixture.

Component	Volume (µl)	Total volume (µl)
Master mix 2X	25	50
Forward primer (10 picomoles/microliter)	2	
Reveres primer (10 picomoles/microliter)	2	
Enzyme	2.5	
Template (DNA or RNA)	5	
WFI	13.5	

**Abbreviations:** WFI: water for injection, µl: microliter.

**Table 3:** The thermal conditions for singleplex and multiplex conventional RT-PCR assays.

Step	Temperature (C°)	Duration	cycle
cDNA synthesis	50	30 minutes	1
Denaturation	95	2 minutes	
Denaturation	95	30 seconds	35
Annealing	50	30 seconds	
Extension	72	2 minutes	
Final extension	72	10 minutes	1

It should be noted that in the multiplex PCR assays, as the number of primer pairs increases, competitive inhibition could potentially lead to a decline in PCR efficiency [26]. In step two, 3 pools of multiplexed primers (comprised of 10 picomoles per microliter of each primer) were used to avoid it. Pool 1 included influenza A/H1N1, A/H3N2, Flu B viruses, and SARS-CoV-2 (N and S genes) primers. Pool 2 contained hRSV, HEVs, and HAdV primers. The last pool was comprised of primers for HPIV1–4. In this step, a primary multiplexed conventional RT-PCR assay containing each primer pool, master mix, and one of the specific viruses as the target in each reaction was used to evaluate whether the primer pools could amplify each targeted virus individually. The thermal conditions (Table 3) and reactions’ components (Table 4) are provided.

**Table 4:** The multiplex conventional RT-PCR reaction mixture.

Component	Volume (µl)	Total volume (µl)
Master mix 2X	25	50
Primer pool	5	
Enzyme	2.5	
Template (DNA or RNA)	5	
WFI	12.5	

**Abbreviations:** WFI: water for injection, µl: microliter.

In step three, a multiplex conventional RT-PCR assay was conducted to check the effectiveness of the primer pools in amplifying multiple viruses in a single reaction. For this purpose, the multiplex conventional RT-PCR assays were performed similarly to the previous step, except that 5 μL of extractions from multiplex infected/co-infected samples were used. The reactions’ components and thermal conditions were similar to the previous step. In all assays, any possible contamination was checked using negative controls.

### Sanger sequencing

Sanger sequencing was carried out to confirm the singleplex conventional RT-PCR assay results as follows: at first, PCR products were purified using the QIAquick High-Prep PCR purification kit (Qiagen, Hilden, Germany), according to the manufacturer’s protocols. Forty nanograms of DNA template (1 µL) and 0.5 µL (10 pmol) specific primer were added to the PCR premix (20 µL final volume). The purified PCR products were sequenced using BigDye Terminator v3.1 Cycle Sequencing Kit (Applied Biosystems, Thermo Fisher Scientific, USA). After that, the resulting products were purified using HighPrep DTR beads (MagBio Genomics, MD, USA). Finally, a 24-channel ABI 3500XL genetic analyzer (Applied Biosystems) was used to sequence purified products.

### Nanopore library preparation and sequencing

**Fig 1:**
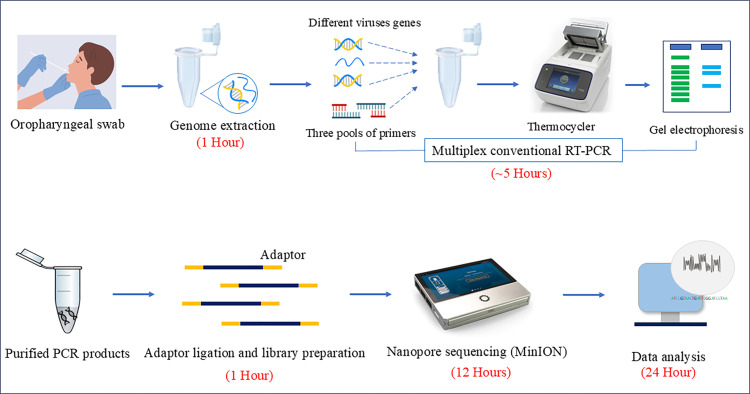
An overview of amplicon-based multiplex nanopore sequencing and turnaround time for SARI-causing virus detection in this study.

The singleplex and multiplex PCR products were purified using a PCR purification kit (Qiagen, Hilden, Germany). The DNA library was prepared using a rapid barcoding kit (SQK-RBK110.96) according to the manufacturer’s instructions (ONT, London, UK). PCR products were pooled together regardless of their yield. In this way, purified amplicons were labeled with specific Rapid Barcodes (RBs). After barcoding, the samples were pooled into a single mixture. The pooled mixture was purified on a magnetic rack and cleaned up using AMPure XP beads in a ratio of 1:1. The Qubit 4.0 fluorometer (Invitrogen, Thermo Fisher Scientific) was used to quantify barcoded PCR products. A proper concentration (800 ng) of the purified library was loaded onto Oxford Nanopore MinION SpotON Flow Cells FLO-MIN106D, R9.4.1 (v9), on the MK1C device (Oxford Nanopore Technologies). The new flow cell contained more than 1200 sufficient functional pores, and the sequencing proceeded for 12 hours using standard settings. The MinKNOW software collected sequencing data and base calls in real-time (Fig 1 shows the protocol’s overall workflow and time).

### Analysis of MinION sequencing data

The quality of the raw data MinION reads and trimming was assessed using Fastp [27]. The FASTQ files were assembled, mapped, and analyzed to generate FASTA files by Medaka [28]. SAMtools v1.20 was employed to calculate the read depth at each position and coverage of the reads [29]. Using NCBI BlastN, the pairwise nucleotide similarity of the consensus sequences to the corresponding reference sequences was evaluated [30,31]. During data analysis, amplicon length differences were considered in sequence mapping and coverage calculations to guarantee accurate detection and quantification of all targeted viruses.

### Assessment of sequence accuracy

To evaluate sequence accuracy, pairwise alignment was performed using ClustalW in BioEdit [32,33]. Herein, the similarity between the consensus sequences generated by Nanopore and those generated by Sanger sequencing was assessed [31,34].

### Specificity of nanopore sequencing for virus detection

To check the specificity of three primer pools in NPS, confirmed positive samples for all viruses were chosen based on the results of conventional RT-PCR and Sanger sequencing tests. Then, in each reaction of the amplification step of NPS, the virus was added as the template, and their primers were absent in that tube. These reactions were tested in duplicate using all three primer pools. Following nanopore sequencing, reads were mapped to reference genomes to determine off-target amplification. Our specificity criteria were as follows: Specificity was considered high if more than 99.9% of sequencing reads failed to map to the genome of the targeted viruses in the absence of specific primers. A minimal percentage of reads (≤0.1%) mapping to the target virus genome was considered acceptable, as low-level background signals can occur due to minor cross-amplification. In addition to primer-specificity testing, negative control reactions (no template control) were included to confirm the absence of contamination.

### Limit of detection (LOD) of NPS

A series of 10-fold dilution gradients (1/10–1/100000) from each viral target extraction was provided to evaluate the LOD. rRT-PCR on all dilutions was performed using the Invitrogen SuperScript III Platinum One-Step Quantitative RT-PCR System (Thermo Fisher Scientific, Waltham, MA, USA). Subsequently, for each target, the main sample and 3 dilutions (original samples, 1/100, 1/1000, and 1/100000) with different Ct values underwent conventional RT-PCR according to the previously mentioned singleplex conventional RT-PCR protocol. As a part of the sensitivity experiment, a negative control was also incorporated. Afterward, the PCR products were analyzed by gel electrophoresis and subjected to nanopore sequencing. Finally, low-quality reads were removed, and the rest were analyzed. We recommended a more conservative approach for establishing the LOD to increase reliability: the highest dilution level that consistently shows visible amplicon bands in gel electrophoresis and meets the criteria of 50% coverage, a minimum read length of 300 base pairs, and a Q30 score greater than 1%. Careful interpretation is advised for any detection at Ct values more than 35, since this may necessitate further replicates or validation using alternative assay techniques.

### Validation of the method

After the test was established, 20 clinical OPS samples with known virus infections or co-infections (samples previously tested by RT-qPCR for detecting the single infection or co-infections) were utilized to evaluate our method. Following the method from extraction to nanopore sequencing analysis to assess if the platform could perform the multiplex detection. For SARS-CoV-2 validation, we targeted only the SARS-CoV-2 N gene, which is highly conserved across various viral strains, making it a reliable target for detection.

### Statistical analysis

To assess the effectiveness of our multiplex detection approach in identifying respiratory viruses, we calculated the positive predictive value (PPV) and negative predictive value (NPV). PPV represents the ratio of true positive results (correctly identified positive cases) to all positive test results. NPV indicates the ratio of true negative results (correctly identified negative cases) to all negative test results. These values were derived using the following formulas:

-
PositivePredictiveValue(PPV)=Truepositive/Truepositive+Falsepositive

-
NegativePredictiveValue(NPV)=Truenegative/Truenegative+Falsenegative

## Results

### Conventional RT-PCR

The selected samples with Ct values under 30 underwent sequencing using NPS. Agarose gel electrophoresis verified the target genes’ amplification in singleplex conventional RT-PCR reactions. The lengths (bps) of PCR products obtained after conventional PCR and gel electrophoresis for Influenza A (H1N1), Influenza A (H3N2), Influenza B, SARS-CoV-2 (N-gene), SARS-CoV-2 (S-gene), HPIV-1, HPIV-2, HPIV-3, HPIV-4, HEV, HAdV, and hRSV as expected were 998 bp, 973 bp, 1069 bp, 1144 bp, 1120 bp, 1102 bp, 1053 bp, 1113 bp, 902 bp, 668 bp, 1298 bp, and 1197 bp, respectively.

### Sanger sequencing

Sanger sequencing and subsequent nucleotide analysis with BLAST confirmed the sequence of the singleplex conventional RT-PCR products and provided an opportunity to compare them with NPS results, except for HPIV-4. The GenBank (www.ncbi.nlm.nih.gov/genbank) accession numbers of the sequences generated from the Sanger sequencing are PQ142371, PQ142372, PQ143318, PQ143319, PQ143300, PQ153873, PQ153872, PQ145194, PQ159148, PQ159149, PQ159150.

### Multiplex nanopore sequencing

**Fig 2:**
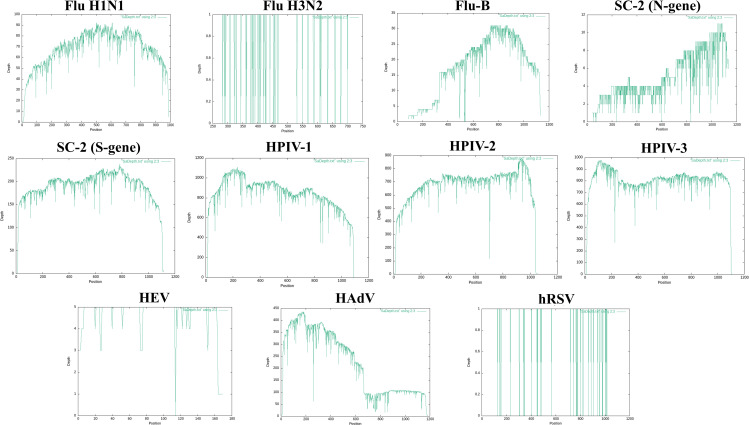
Coverage map of gene targets for SARI-related viruses obtained from pooled samples in the Nanopore sequencing method. Abbreviations: Flu: Influenza virus, HPIV: human parainfluenza virus, HEV: human enteroviruses, HAdV: human adenovirus, hRSV: human respiratory syncytial virus.

The products of singleplex and multiplex conventional RT-PCR reactions (except for HPIV-4) were sequenced by the Oxford nanopore platform using the MinION device. Our criteria for positive virus detection were defined as meeting at least two of these parameters: 1) a mean read length of at least 300 bp, 2) a coverage of more than 50%, and 3) a Q30 value of more than 1%. Given our objective to develop a multiplex detection method for respiratory viruses, we established a coverage threshold of 50% to ensure the effective identification of target viruses while maintaining sequencing capacity with the MinION device. Additionally, we focused on the targeted detection of specific respiratory viruses rather than whole-genome sequencing. As the range of our amplicons was 668 bp to 1298 bp, a 50% coverage criteria means that each amplicon contains at least 334 bp to 649 bp of sequence data. Although we accept that a 10%–20% sequencing depth and 90% coverage threshold would further increase specificity, this was adequate for confident detection. With an average of 515.8 Mb of total reads per flow cell, all samples were successfully amplified and sequenced utilizing the nanopore sequencing assay methodology. Bioinformatic analysis showed that a total of 711,000 reads passed the quality filters (Q-score≥7). Q-scores less than 7 were excluded from the analysis. Most of the samples produced 90−100% coverage. Samples with higher Ct values showed a higher frequency of drops in coverage. The initial viral loads in the clinical samples varied, as reflected in their respective Ct values from RT-qPCR. Lower viral loads resulted in fewer amplicons, leading to lower sequencing coverage (e.g., HEV). Conversely, higher viral loads contributed to more abundant sequencing reads (e.g., HAdV) (Fig 2 shows the coverage map). The Q30 value for all the viruses was above 1%. In this experiment, HAdVs and HPIV-2 with 100% and HEVs with 22.3% had the highest and the lowest coverages, respectively. The negative control sample did not produce any false-positive readings. The multiplexed sequencing data’s statistics of viral reads in each barcoded sample were nearly identical to those of the data from the single individual run sample (the data of multiplex nanopore sequencing was not shown). The method could detect and type all viruses in samples according to the mapping of the reads to the corresponding genome reference sequences. Our comprehensive comparison of RT-PCR and NGS findings shows that most discovered targets have good concordance. The raw sequencing data produced by this investigation has been submitted to NCBI SRA with accession number PRJNA1145989. The run metrics provided in Table 5.

**Table 5:** Summary of MinION sequencing statistics for the method establishment.

Virus	Ct value	Total data yielded (kb)	Number of reads	Mean read length (bp)	Total bases	Total bases aligned	Coverage^1^	Reads >Q30^2^ (%)
SARS-CoV-2 N-gene	24	2951.526	4.000000 K	649.7	1.805870 M	5,680.0	94.48	1.98
SARS-CoV-2 S-gene	20	2650.178	4.000000 K	579.0	1.427962 M	208,494.0	99.10	7.47
H1N1	23	2273.965	4.000000 K	536.1	1.128672 M	69,652.0	98.6	2.58
H3N2	27	490.618	660	528.0	307.420000 K	405.0	40.5	1.06
Flu-B	26	2276.597	3.063000 K	663.8	841.367000 K	19,279.0	60	6.62
HAdVs	20	3055.912	4.000000 K	888.1	1.805651 M	272,124.0	100	6.26
HEVs	29	2911.363	4.000000 K	300.0	1.660309 M	813.0	22.3	6.24
hRSV	25	3156.429	4.000000 K	1,219.0	1.885557 M	990.0	81	7.28
HPIV-1	22	2847.921	4.000000 K	559.6	1.605978 M	939,512.0	99.18	6.94
HPIV-2	21	2674.119	4.000000 K	546.3	1.439296 M	724,338.0	100	6.68
HPIV-3	21	2941.321	4.000000 K	581.0	1.683752 M	904,749.0	99.20	7.86
NC	–	–	–	–	–	–	–	–

All the viruses, except HPIV-4, passed the detection criteria and could be identified by this platform. **Abbreviations:** SARS-CoV-2: Severe acute respiratory syndrome coronavirus 2, Flu-B: Influenza-B, HAdV: human adenovirus, HEVs: human enterovirus, hRSV: human respiratory syncytial virus, HPIV: human parainfluenza virus, NC: negative control, Kb: kilobase, M: million, bp: base pair. ^1^ Coverage refers to the average number of reads at each position in a genome; ^2^ Q30 value means every 1000 bp sequencing read may contain an error.

### Assessment of sequence accuracy

Comparing sequences obtained from the NPS and the Sanger method demonstrated high nucleotide identity using the ClustalW algorithm in the Bioedit software (Figures in S1–S11 Figs).

### Specificity of NPS for virus detection

To evaluate the specificity, the target viruses were added to the reaction mixes that lacked the specific primers. For each virus we checked the specificity. We analyzed the obtained sequences based on that described in “Analysis of MinION sequencing data” section. For each virus, more than 99.9% of reads did not map to the genome of the targeted viruses. However, the remaining reads (0.1%) were mapped to the targeted viruses.

### Limit of detection (LOD) of NPS

**Fig 3:**
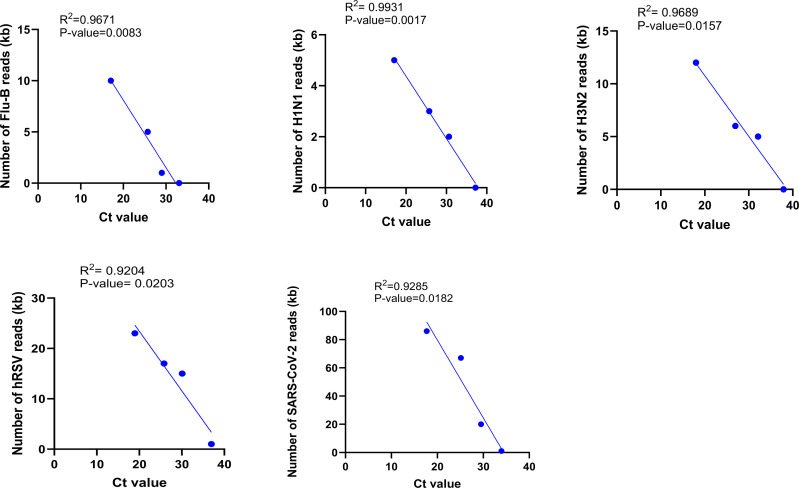
Correlation between the Ct value and the total number of SARI-related viruses generated reads in the MinION sequencing. All correlations were significant (P-value<0.05). Abbreviations: Flu-B: Influenza-B, hRSV: human respiratory syncytial virus, Kb: kilobase.

According to our results, as the initial viral loads dropped in the sample dilutions, the visibility of amplicon bands in PCR electrophoresis and the number of generated reads in the nanopore sequencing decreased. Utilizing serial dilutions of known positive samples for SARI-related viruses, the rRT-PCR Ct values of 33.9, 33.05, 37, 37.9, and 36.9 were determined as LOD for SARS-CoV-2, Flu B, Flu A/H1N1, Flu A/H3N2, and hRSV detection, respectively. The results represented a significant negative correlation between the Ct value of rRT-PCR and the number of nanopore sequencing reads for each virus (p-value<0.05) (Fig 3).

No read was detected in negative controls. For the HAdV and the HEVs LOD determination, the nanopore sequencing did not yield sufficient data to meet our positive detection threshold. At the time of the study performance, the available sample for HAdV had a high Ct value. We could not obtain a high-titer sample with a lower Ct value, which limited our ability to determine this virus’s LOD accurately. For HEVs, a fresh clinical sample was unavailable. Accordingly, we had to rely on a frozen extracted RNA, which might have affected amplification efficiency, sequencing performance, and the ability to determine the LOD. The limitations show that more research involving high-quality samples and high viral load is required to further improve the detection limits for these viruses. Moreover, for HPIVs, because of the lack of positive clinical samples, we could not determine the LOD for them.

### Validation of the method

In this step, we chose samples previously tested by RT-qPCR for the presentation of a single infection or co-infection (Ct values are shown in Table 6). Using the NPS, we could successfully detect the respiratory viruses in the 20 known positive SARI samples, in which 15 samples were positive for one virus, and five were probably positive for two viruses simultaneously (Table 6). Notably, samples with lower Ct values in rRT-PCR showed higher coverage in NPS. Unfortunately, we could not identify more co-infections in our samples using RT-qPCR due to a small sample size and lack of co-infected clinical samples. As a result, we could not identify other probable co-infections using the nanopore sequencing method.

**Table 6:** Summary of MinION sequencing reads statistics for the method validation.

Virus		Ct value	Total data yielded (kb)	Number of reads	Mean read length (bp)	Total bases	Total bases aligned	Coverage^1^	Reads>Q30^2^(%)
SARS-CoV-2 (N gene)		27	1105.726	1.876000 K	573.3	581.863000 K	1,020.0	41.85	2.2
SARS-CoV-2 (N gene)		23	714.873	1.174000 K	759.5	389.112000 K	1,459.0	80.2	1.61
SARS-CoV-2 (N gene)		25	2143.943	2.877000 K	558.8	1.353136 M	2,217.0	72.5	1.38
H1N1		27	220.523	409	419.0	103.851000 K	325.0	31.5	4.7
SARS-CoV (N gene)-2		21	2369.246	3.134000 K	701.4	1.499904 M	3,588.0	90.38	1.08
Flu-B/hRSV co-infection	hRSV	30	2742.125	4.000000 K	576.0	1.621822 M	225.0	18.03	2.05
	Flu-B	29	2725.492	4.000000 K	851.0	1.606046 M	563.0	24.22	1.61
Flu-B		27	3486.067	4.000000 K	757.9	2.369260 M	1,871.0	31.70	1.53
Flu-B/SARS-CoV-2co-infection	Flu-B	30	1035.110	1.971000 K	300.0	451.786000 K	297.0	11	4.67
	SARS-CoV-2 (N gene)	26	1035.110	1.971000 K	418.5	451.786000 K	2,328.0	50.08	4.67
SARS-CoV-2 (N gene)		26	988.649	1.922000 K	412.4	420.478000 K	1,366.0	46.1	5.09
H1/hRSV co-infection	H1N1	24	2685.944	4.000000 K	869.0	1.503742 M	643.0	61.7	2.15
	hRSV	27	2807.680	4.000000 K	1,098.0	1.629700 M	438.0	35.80	2.20
H1N1		22	2556.438	4.000000 K	813.2	1.378335 M	1,549.0	75.07	2.19
Flu-B		27	3104.067	4.000000 K	697.8	1.924041 M	2,584.0	27.8	1.92
Flu-B		29	2807.757	4.000000 K	591.5	1.639052 M	575.0	21.14	2.14
SARS-CoV-2 (N gene)		21	2465.879	4.000000 K	739.7	1.287492 M	1,422.0	83.3	2.46
SARS-CoV-2 (N gene)		20	2490.328	4.000000 K	710.7	1.316322 M	7,312.0	92.46	2.31
SARS-CoV-2 (N gene)		19	3111.436	4.000000 K	690.6	1.939042 M	7,086.0	95.40	1.59
SARS-CoV-2 (N gene)		20	3101.367	4.000000 K	733.0	1.927898 M	22,571.0	95.50	1.90
HPIV-1		25	2468.899	4.000000 K	1,125.0	1.539252 M	749.0	65.9	1.54
HPIV-1/HPIV-3 co-infection	HPIV-1	23	2937.305	4.000000 K	601.0	1.799928 M	921.0	72.8	1.59
	HPIV-3	26	1777.735	3.290000 K	660.0	818.045000 K	574.0	52	5.63
hRSV/HAdV co-infection	hRSV	21	3156.429	4.000000 K	1,219.0	1.885557 M	990.0	80.05	7.28
	HAdV	19	2324.212	4.000000 K	1,245.0	1.145664 M	1,136.0	94	4

This method could detect 15 single and 5 probable co-infections in the known positive OPS samples. For SARS-CoV-2 we targeted only the N gene for validation. **Abbreviations:** SARS-CoV-2: Severe acute respiratory syndrome coronavirus 2, Flu-B: Influenza-B, HAdV: human adenovirus, hRSV: human respiratory syncytial virus, HPIV: human parainfluenza virus, Kb: kilobase, M: million, bp: base pair. ^1^ Coverage refers to the average number of reads at each position in a genome; ^2^ Q30 value means every 1000 bp sequencing read may contain an error.

### Statistical analysis

The PPV value for all the viruses in this method was 100%, as we had no false positive results. But, the NPV value was variable among different viruses. The NPV for Influenza A (H1N1), Influenza A (H3N2), Influenza B, SARS-CoV-2 (N-gene), SARS-CoV-2 (S-gene), HPIV-1, HPIV-2, HPIV-3, HEV, HAdV, and hRSV were, 83.3%, 100%, 100%, 85.7%, 88%, 100%, 100%, 100%, 82.5%, 100%, 97.2%, respectively.

## Discussion

Rapid clinical diagnosis and outbreak surveillance are two specialized areas where NPS has been used [35]. The popularity of this platform for diagnosis is growing due to its portability and ease of use, with no need for momentous initial investment, and the possibility of real-time data analysis [9,36]. One of the most critical aspects of the prevention and management of SARI cases is rapid pathogen identification [37]. The multiplex nanopore sequencing technique, using specific barcodes, makes it possible to sequence several samples at once. Multiplexing strategies provide benefits such as increased output, improved cost efficiency, and shortened sequencing run times [14]. However, cross-sample contamination may result in erroneous results interpretation and data inaccuracies [38]. The recent emergence of SARS-CoV-2 and probable epidemics and pandemics of other respiratory pathogens in the future reveal the importance of keeping an eye on the circulation and even co-circulation of respiratory viruses and being prepared to respond quickly to both domestic and international public health threats [13].

To expand the range of NPS-based virus detection, this study developed a protocol that combines the benefits of multiplex PCR’s “high specificity and simultaneous detection” with NPS’s “high sensitivity and high throughput” for detecting respiratory viruses, including SARS-CoV-2, Flu A and B, hRSV, HAdVs, HEVs, and HPIV1–3 in SARI cases. We pooled libraries and ran multiplex MinION sequencing. Analysis of the sequencing data presented 515.8 Mb of total reads. For each sample, the amplicon sequencing resulted in more than 1% of the Q30 value and more than 300 bp mean read length. Most of the respiratory viruses’ reads had higher than 90% coverage compared to the corresponding reference sequences. The differences in coverage caused by multiplex techniques had been a disadvantage since areas with poor coverage also had decreased sequence accuracy. According to our findings, consensus sequences were most accurate when they were called from regions with more coverage. Our data analysis showed that this method could successfully identify all the targeted viruses (except HPIV-4) detected by singleplex rRT-PCR in advance. The PPV value for each virus detection using this method was 100%. But, the NPV value was vary among different viruses. For Influenza A (H1N1), Influenza A (H3N2), Influenza B, SARS-CoV-2 (N-gene), SARS-CoV-2 (S-gene), HPIV-1, HPIV-2, HPIV-3, HEV, HAdV, and hRSV the NPV value were, 83.3%, 100%, 100%, 85.7%, 88%, 100%, 100%, 100%, 82.5%, 100%, 97.2%, respectively.

The variation in amplicon size was carefully considered when designing the primer and has no negative impact on sequencing performance for some reasons. A) Compatibility with Nanopore Sequencing: The MinION platform handles various fragment sizes without showing significant bias. Long amplicon sequencing is suitable for the ONT. Previous studies have demonstrated that mixed-length amplicons in multiplex applications can be handled effectively by nanopore sequencing [11,39]. B. Balanced Pooling Strategy: Before library preparation, we optimized amplicon pooling to ensure optimal sequencing efficiency. While normalization guarantees equimolar representation, sequencing efficiency is not entirely equalized. Although a crucial step in ensuring balanced representation is normalizing amplicon concentrations before sequencing, obtaining a 100% consistent sequencing result is difficult because of some factors that affect ultimate sequencing efficiency. Amplification biases in PCR can result in preferential amplification of some targets over others, even after normalization. Several variables, including secondary structures, GC content, and primer binding efficiency, can affect how various amplicons are represented [40,41]. Variations in sequencing efficiency can be caused by secondary structures, GC concentration, and amplicon length. Longer amplicons or those with a high GC content may be less effective at nanopore sequencing, according to studies [42–45]. The effectiveness of RT-PCR amplification is influenced by the quality of the extracted viral genome. Even after normalization, clinical samples with low input RNA quantities or damaged viral RNA may still produce weaker amplicons, which could result in decreased sequencing efficiency [46]. C. Bioinformatics Analysis Adjustments: To ensure precise detection and quantification of all targeted viruses, amplicon length differences were considered during data analysis in sequencing mapping and coverage calculations.

Our panel did not include H5N1 for some reasons. Initially, we focused on the respiratory viruses most frequently linked to SARI in our area (Iran). Even though H5N1 is a recognized zoonotic disease with the potential to spread like a pandemic, there haven’t been many cases of persistent human infections in Iran in recent years [47–49], With sporadic detections in birds rather than widespread human cases. Our diagnostic panel was created to address the most clinically relevant respiratory viruses affecting human populations, but H5N1 inclusion was not a top priority. Because of Iran’s low number of H5N1 human cases, we prioritized viruses that are more common and significantly influence public health in our area. However, our nanopore sequencing method is still flexible, and H5N1 can be included in subsequent research, mainly if its epidemiological significance in Iran grows. Continuous surveillance is essential to expand the panel and detect emerging threats, including avian influenza viruses, which could be valuable for future research.

Nanopore sequencing has been applied in former studies to identify viral infectious pathogens in real time in clinical samples [50–54]. In the amplicon-based nanopore sequencing study for the Hantaan virus (HTNV) sequencing, researchers found that for the L, M, and S segments, the HTNV genome coverage was 65.4–96.8%, 90.6–98.5%, and 92.1–100%, respectively [9]. Wang et al. created a high-throughput sequencing technique based on nanopore targeted sequencing. They found 34 positive samples out of 45 suspected samples, which detected 15 cases more than RT-qPCR detection [8]. For multiplex detection, a study of 96 suspected samples of arbovirus infection showed that a single assay by NPS could accurately identify the different viruses in 83.67% (41/49) of the clinical samples [25].

To assess the sensitivity of our NPS method, the results revealed that for SARS-CoV-2, Flu A/H1N1, Flu/A H3N2, Flu B, and hRSV, the maximum Ct values for trustworthy detection, which produced sufficient data with proper coverage, ranged from 33.05 to 37.9. It became clear that when Ct values increased, the number of mapped reads trended downward. Other studies have previously reported this negative correlation [25,55]. Crossley et al. used ONT sequencing to determine the LOD for avian influenza viruses by evaluating sequencing depth and detection consistency across varying viral loads. The dilution series was made to cover the MinION sequencing limit of detection by incorporating a variety of matrix gene rtPCR Ct values (16). A study by Lewandowski et al. established LOD for metagenomic nanopore sequencing of influenza viruses by correlating viral load (Ct) with the number of sequencing reads (17). Another study evaluated targeted NGS for detecting bovine pathogens and compared detection thresholds with traditional diagnostic methods. The NGS method was able to identify the organisms from samples with qPCR Cycle Threshold (CT) values in the 30s. The detection limits of the targeted NGS method were assessed using ten representative pathogens that were also examined by qPCR (18). Our primers were designed to target highly conserved regions, which increases amplification efficiency, and PCR cycling was optimized to amplify even low-viral-load samples before sequencing. Despite successful amplification at Ct levels of 37 and above for certain viral targets, we recognize that sequencing efficiency and depth at this level may not be consistently reliable. To ensure greater reliability, we suggest a more cautious approach for defining the LOD: the highest dilution level that consistently shows visible amplicon bands in gel electrophoresis and meets the criteria of 50% coverage, a minimum read length of 300 base pairs, and a Q30 score above 1%. Any detection at Ct values above 35 should be interpreted cautiously, as it may require additional replicates or validation using other assay methods. In future research, we aim to increase the sequencing depth to improve read recovery from high Ct samples. We detected HPIV1–3 in SARI OPS sample detection; meanwhile, due to the lack of clinical samples, we could not determine the LOD level. Moreover, our LOD determination data for HAdV and HEVs did not meet the criteria for positive detection. The specificity evaluation of our NPS test showed this method identified mentioned SARI-related viruses in given clinical samples with 99.9% specificity. The avian Flu A detection by multiplex NPS presented 83% sensitivity and 100% specificity [56]. Regarding SARS-CoV-2 LOD determination, our result was similar to the study of multiplex nanopore sequencing for different viral and bacterial pathogen detections (Ct value up to 33) [57]. In identifying avian IAVs, based on detecting all IAV segments with high coverage depth, researchers determined the Ct value of 35 as the LOD of their method [56]. Notably, the LOD of our test for NTS showed a non-inferior performance when compared to the detection limits of other nanopore sequencing assays from existing kinds of literature. However, more testing could help assess the precise detection limit.

Up to now, the most common test to identify viral co-infections has been real-time PCR. Studies revealed significant variations in the prevalence of respiratory virus co-infections [58–61]. The importance of co-infections and their correlation with the severity of respiratory diseases has also been controversial [62–64]. Nonetheless, early viral co-infection diagnosis can save unnecessary antibiotic use and improve patient care and prognosis [62]. Consequently, the development and increasing efficacy of multi-pathogen diagnostic techniques have been of imperative health concern [65]. In recent years, multiplex nanopore sequencing has been developed and used for multi-pathogen detection at once [8,9,14,25]. In the presented study, the NPS protocol was validated for single infection or probable co-infection detection of SARI-causing viruses. Accordingly, it could successfully identify 10 single- and 5 probable co-infections among SARI OPS samples. Since the SARS-CoV-2 N gene is highly conserved across different virus strains and is a reliable target for detection, we only targeted this gene for SARS-CoV-2 validation. The co-infection cases identified in this study were based on our initial detection threshold (≥50% coverage, a minimum read length of 300 base pairs, Q30 > 1%) and should be interpreted with caution. We aimed to include a dual-criteria approach (e.g., 90% coverage) in future work to enhance specificity and reliability in detecting co-infections. Moreover, in future research, we suggest combining quantitative validation with the ddPCR or other high throughput sequencing like metagenomic sequencing to strengthen our findings. Although we did not include positive samples for common HCoVs, such as HCoV-229E, HCoV-NL63, HCoV-OC43, or HCoV-HKU1, we trust that their presence in the clinical samples could not pose a risk of cross-reactivity or interference with SARS-CoV-2 detection in our panel due to the following reasons: Our assay’s primers were designed to specifically target specific areas of the SARS-CoV-2 genome, minimizing the possibility of cross-reactivity with other coronaviruses. We also performed sequence alignments before primer selection to ensure that primer-binding sites were specific to SARS-CoV-2 and would not have any overlaps with common HCoVs.

RT-qPCR is still the gold standard for respiratory virus detection due to its higher sensitivity, lower detection limits, and well-established clinical validation. RT-qPCR provides rapid results within a few hours, making it ideal for clinical diagnostics where timely detection is critical. While offering multiplexing and sequencing-based advantages [25,31,66], NPS requires longer processing times and higher costs, making it less practical for routine diagnostics. Although nanopore sequencing provides additional benefits, such as detecting co-infections and providing genomic insights beyond simple presence/absence detection, it can’t replace the RT-qPCR as a diagnostic tool.

As a final point, it should be mentioned that we had some limitations. One of the main limitations of this study is the relatively small sample size, which may affect the generalizability of our findings. The availability of clinical samples and study requirements limited our sample size. Because of the small sample size, we couldn’t detect other co-infections in our method validation. Future studies should aim to include a larger and statistically powered of clinical samples to enhance the detection and assess the method’s performance across a broader range of samples. Although in this study, we selected regions for primer design that have remained unchanged over the years, it may not fully capture recombinant or novel viruses with significant genomic changes. Furthermore, while we initially set 50% coverage as a minimum threshold, we acknowledge that 90% coverage criteria may enhance specificity. Future studies can incorporate a dual-criteria Approach: 1) Primary detection threshold: Maintain 50% coverage for initial identification (Ensures sufficient reads to confirm the presence of a target virus and balances sensitivity and sequencing capacity in multiplex settings). 2) High-confidence detection: 10%–20% sequencing depth and 90% coverage are necessary for variant analysis or clinical assessment (Ensures robust identification for variant analysis) [39]. Future research will investigate optimal sequencing depth to increase detection rates for samples with high Ct values. Another limitation was a failure to identify HPIV-4 and the LOD determination for HPIV1–3, HAdV, and HEVs, which could be determined in future investigations. Besides, in LOD determination, detection at Ct values above 35 should be interpreted with caution, as it may necessitate further replicates or confirmation through alternative assay methods. While this method’s current version has limitations, we remain enthusiastic about the possibility of future advancements in this approach and the expansion of the workflow to other viruses. This study proposes a proof of concept for a platform that allows the multiplex detection of respiratory viruses by pooling various amplicons under ONT native barcodes.

## Conclusion

This article presented an approach for the multiplex detection of viruses causing SARI using nanopore next-generation sequencing. These findings provide insights into broad virus detection platforms, particularly during outbreaks. As our current procedure and interpretation of sequencing data have not matured yet, this method requires more advancements.

## Supporting information

S1 Fig SARS-CoV-2 (N-gene) Assessment of sequence accuracy results. (TIFF)

S2 Fig SARS-CoV-2 (S-gene) Assessment of sequence accuracy results. (TIFF)

S3 Fig Influenza A (H1N1) Assessment of sequence accuracy results. (TIFF)

S4 Fig Influenza A (H3N2) Assessment of sequence accuracy results. (TIF)

S5 Fig Influenza B Assessment of sequence accuracy results. (TIF)

S6 Fig HAdV Assessment of sequence accuracy results. (TIFF)

S7 Fig hRSV Assessment of sequence accuracy results. (TIFF)

S8 Fig HEV Assessment of sequence accuracy results. (TIF)

S9 Fig HPIV-1 Assessment of sequence accuracy results. (TIFF)

S10 Fig HPIV-2 Assessment of sequence accuracy results. (TIFF)

S11 Fig HPIV-3 Assessment of sequence accuracy results. (TIF)

## Abbreviations

SARI: Severe acute respiratory infection
WHO: World Health Organization
HCoVs: human coronaviruses
hRSV: human respiratory syncytial viruses
HEVs: human enteroviruses
HAdVs: human adenoviruses
HPIVs: human parainfluenza viruses 1–4
PCRs: polymerase chain reactions
NGS: next-generation sequencing
TGS: Third-generation sequencing
ONT: Oxford Nanopore Technologies
SMRT: single-molecule real-time
NTS: Nanopore-targeted sequencing
SARS-CoV-2: severe acute respiratory syndrome coronavirus-2
OPS: oropharyngeal swab
BLAST: Basic Local Alignment Search Tool
HA: hemagglutinin
N: nucleocapsid
S: spike
HN: hemagglutinin-neuraminidase
NCR: noncoding region
F: fusion
NC: negative control
Kb: kilobase
M: million
bp: base pair
LOD: Limit of detection
HTNV: Hantaan virus
NAT: nucleic acid test
IAV: Influenza A virus
