# Supplementary figures and images for: A targeted approach for multiplex detection of respiratory viruses in cases with severe acute respiratory infections by nanopore sequencing
Source: PLoS One. 2025 Jun 25;20(6):e0324601. doi: 10.1371/journal.pone.0324601 (PMC12192076; doi:10.1371/journal.pone.0324601)

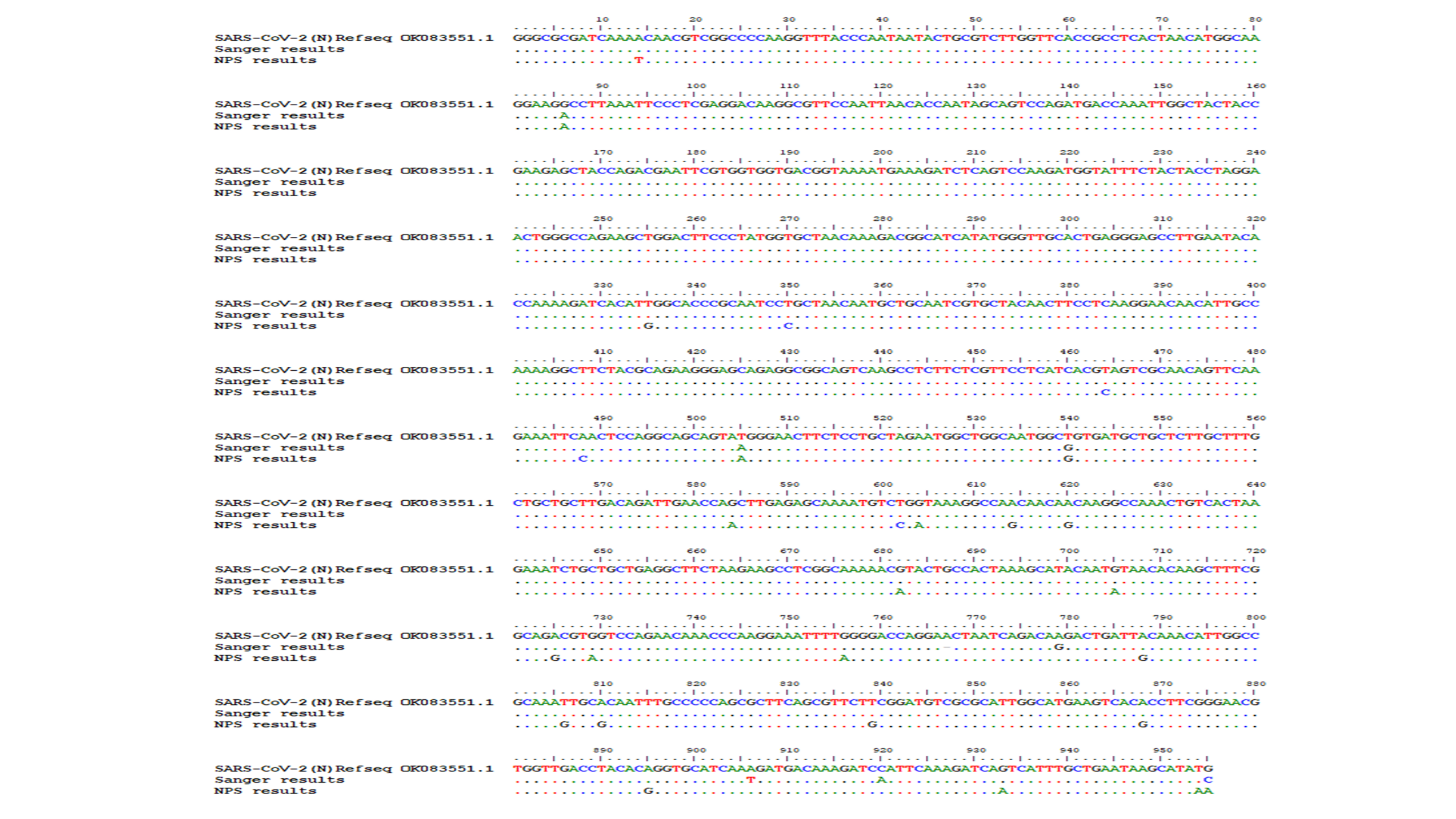

Supplement: S1 Fig — (TIFF) [file pone.0324601.s001.tiff]

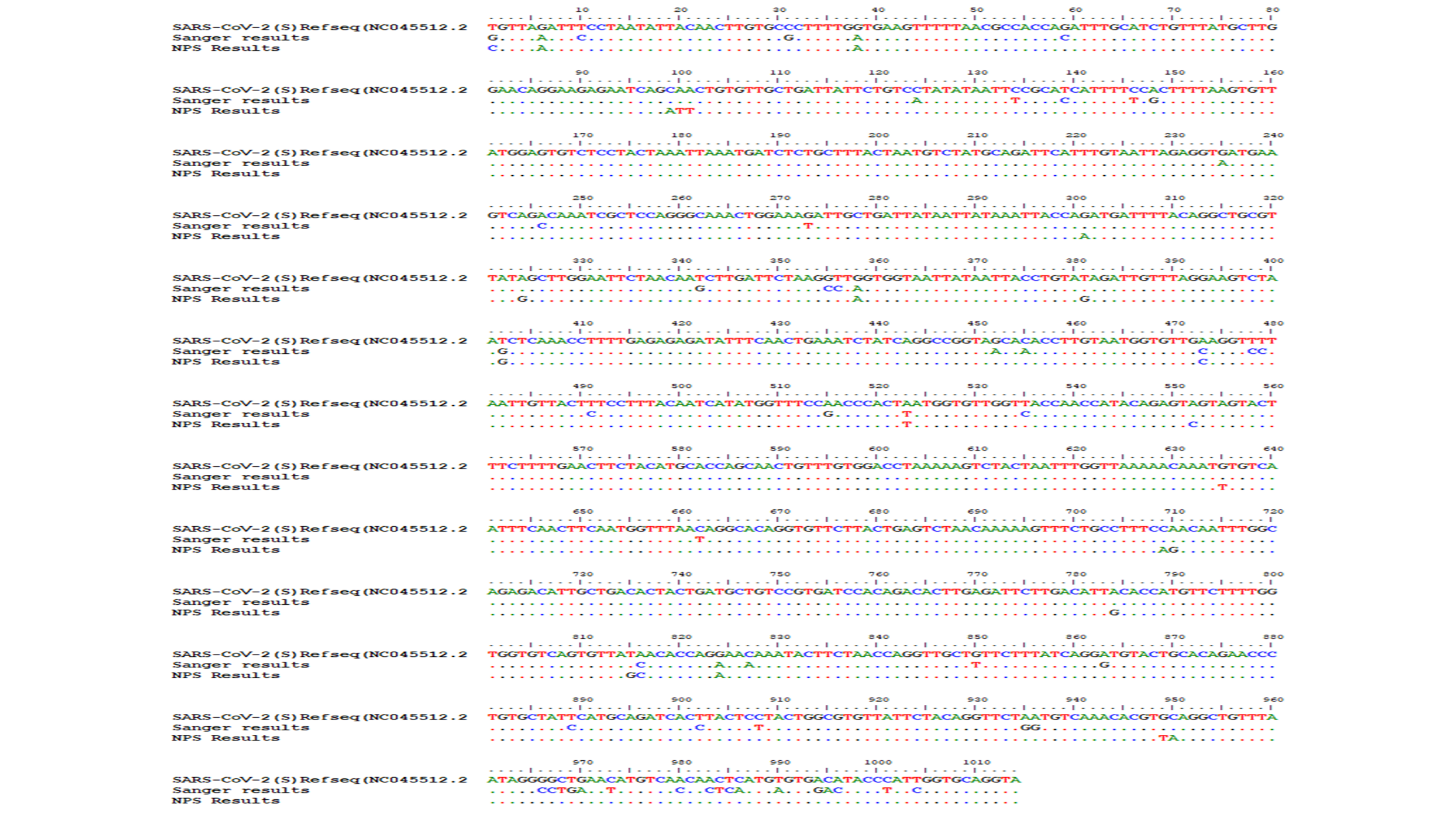

Supplement: S2 Fig — (TIFF) [file pone.0324601.s002.tiff]

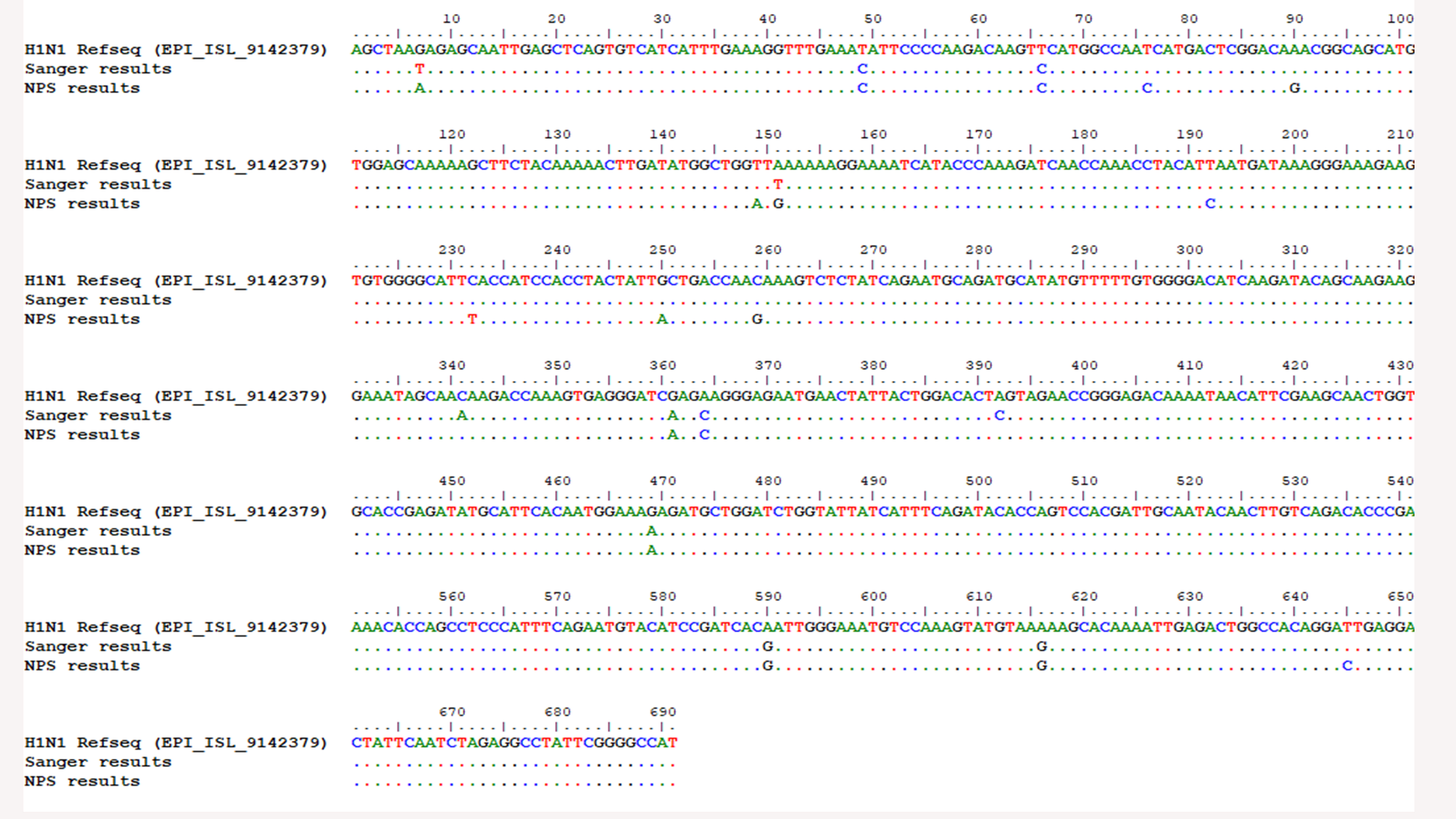

Supplement: S3 Fig — (TIFF) [file pone.0324601.s003.tiff]

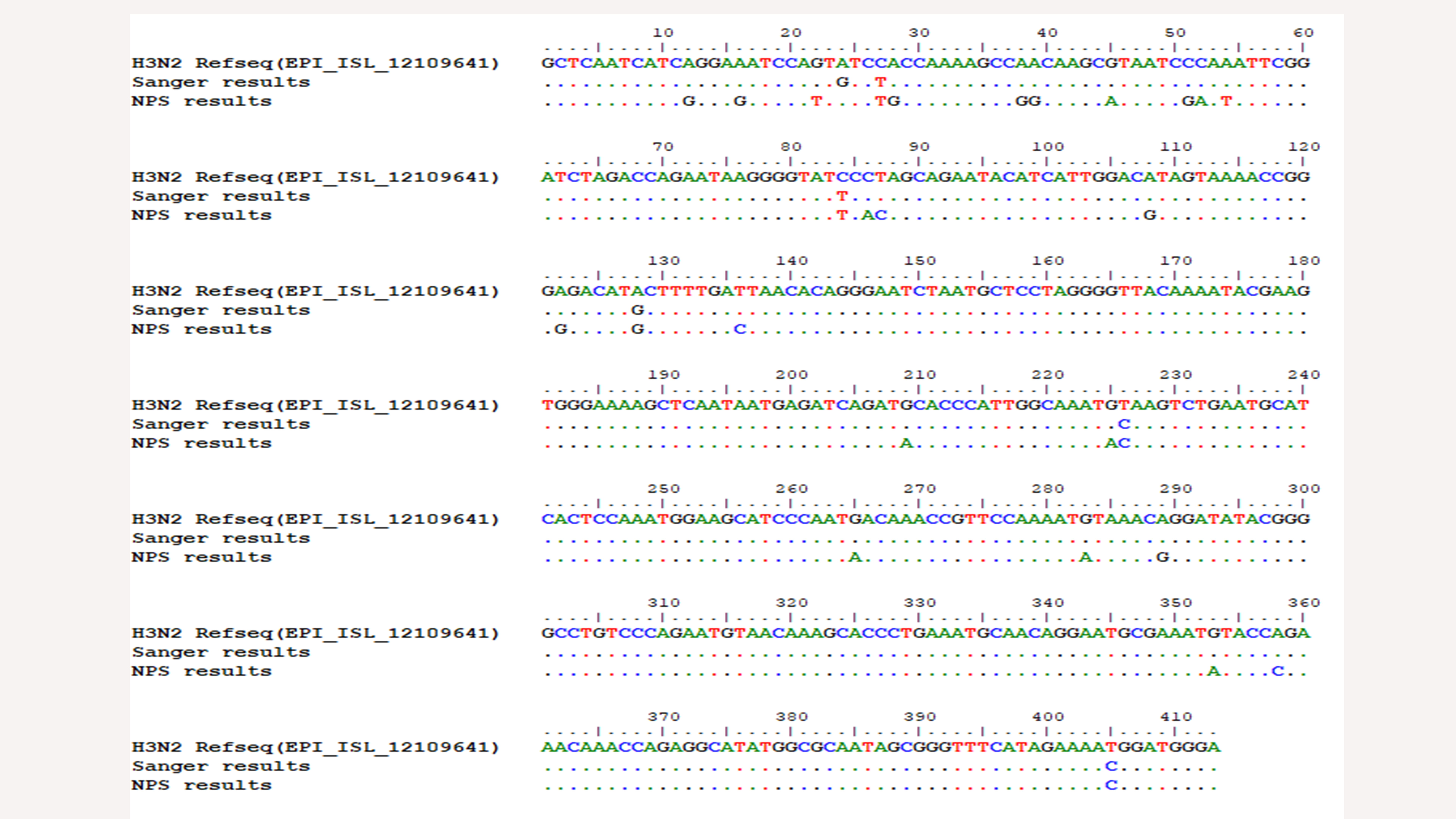

Supplement: S4 Fig — (TIF) [file pone.0324601.s004.tif]

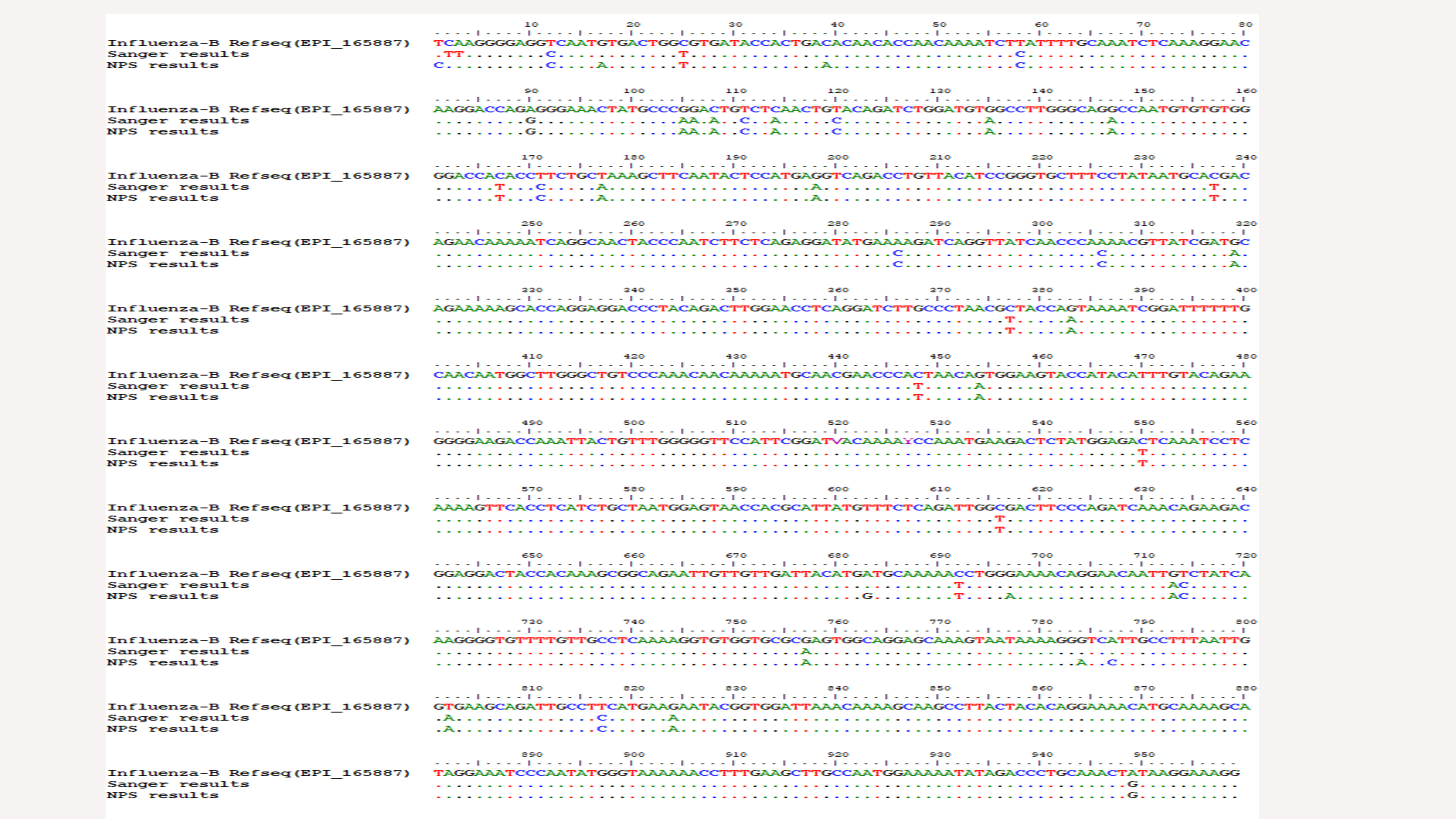

Supplement: S5 Fig — (TIF) [file pone.0324601.s005.tif]

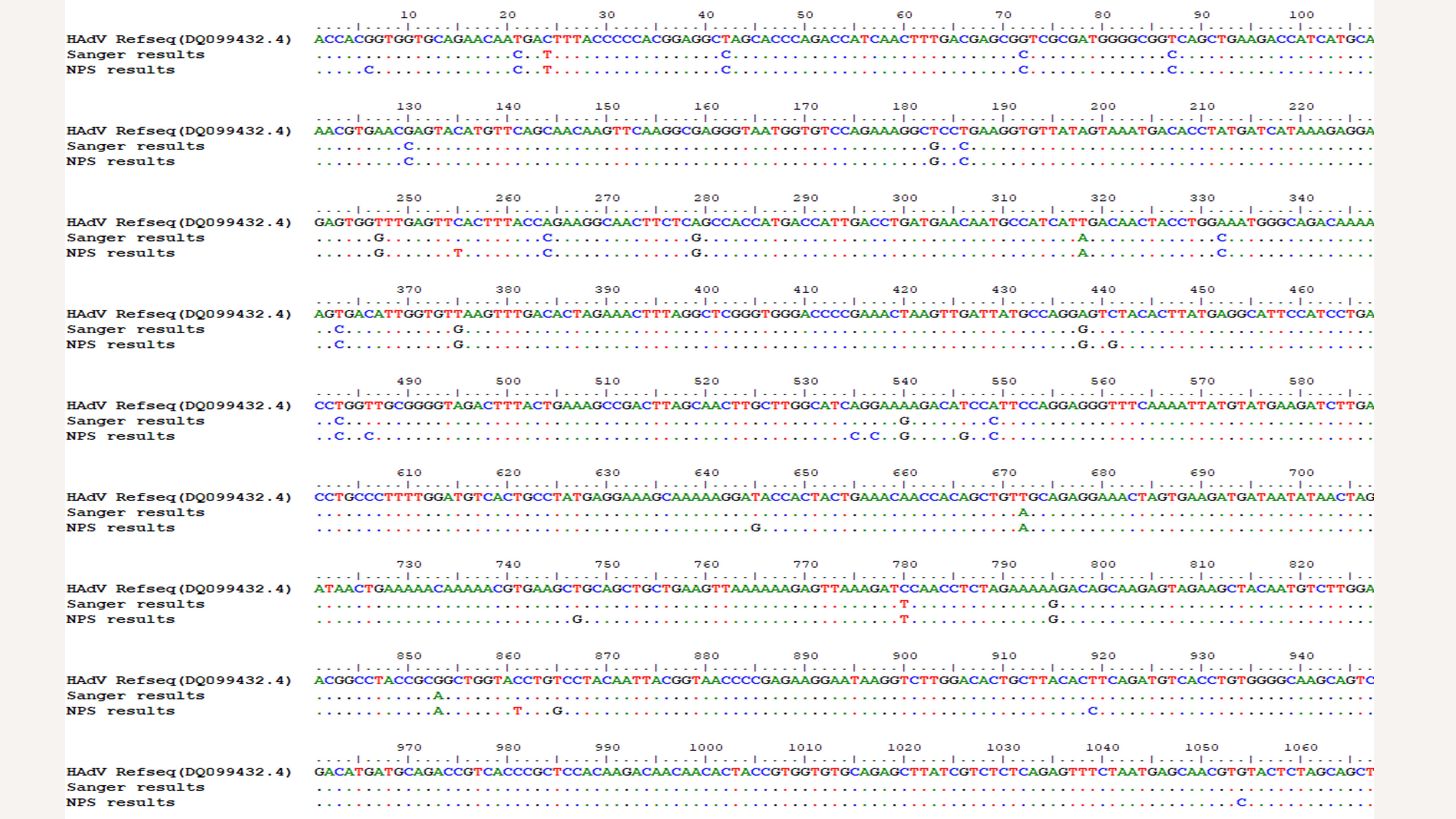

Supplement: S6 Fig — (TIFF) [file pone.0324601.s006.tiff]

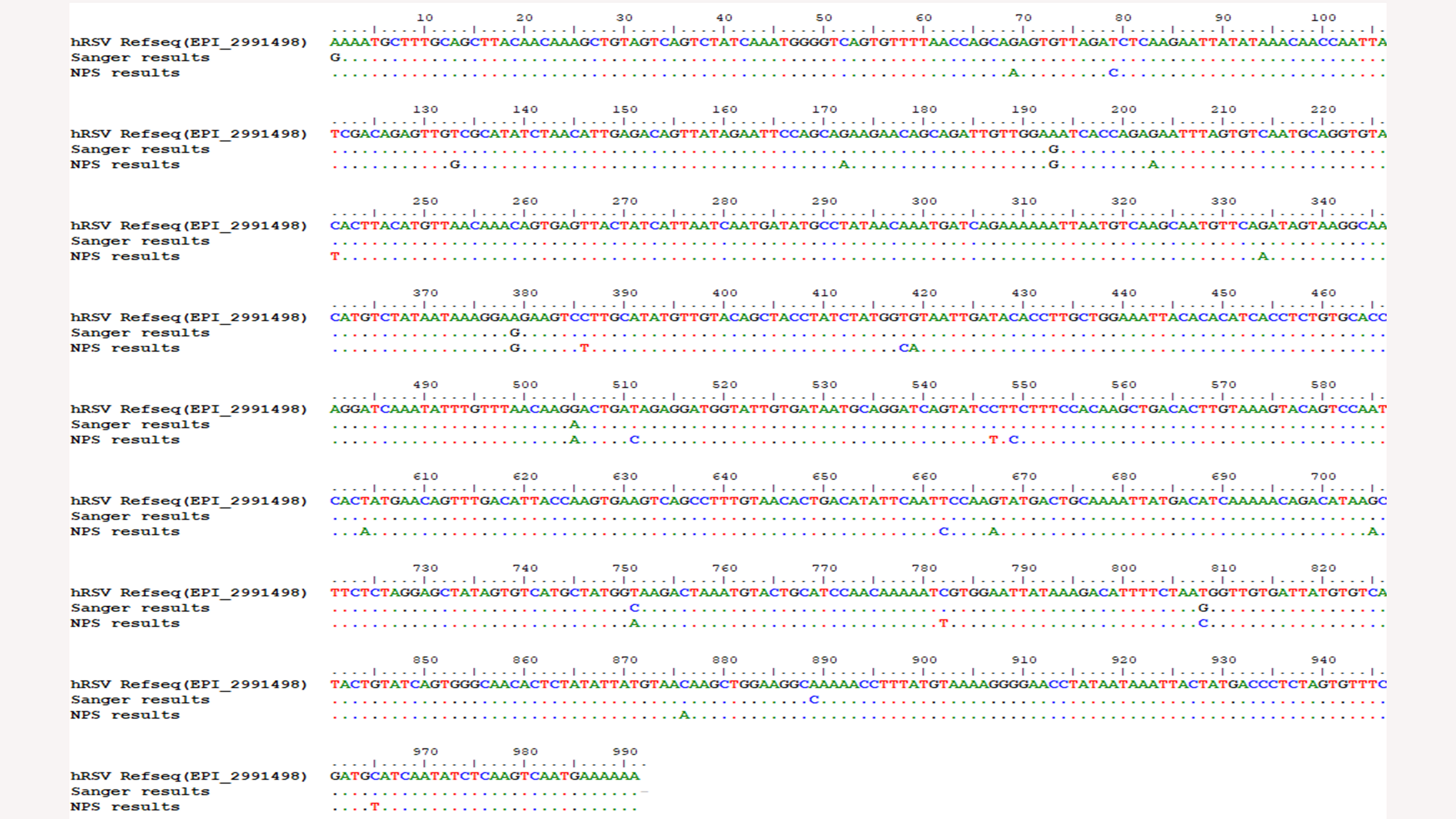

Supplement: S7 Fig — (TIFF) [file pone.0324601.s007.tiff]

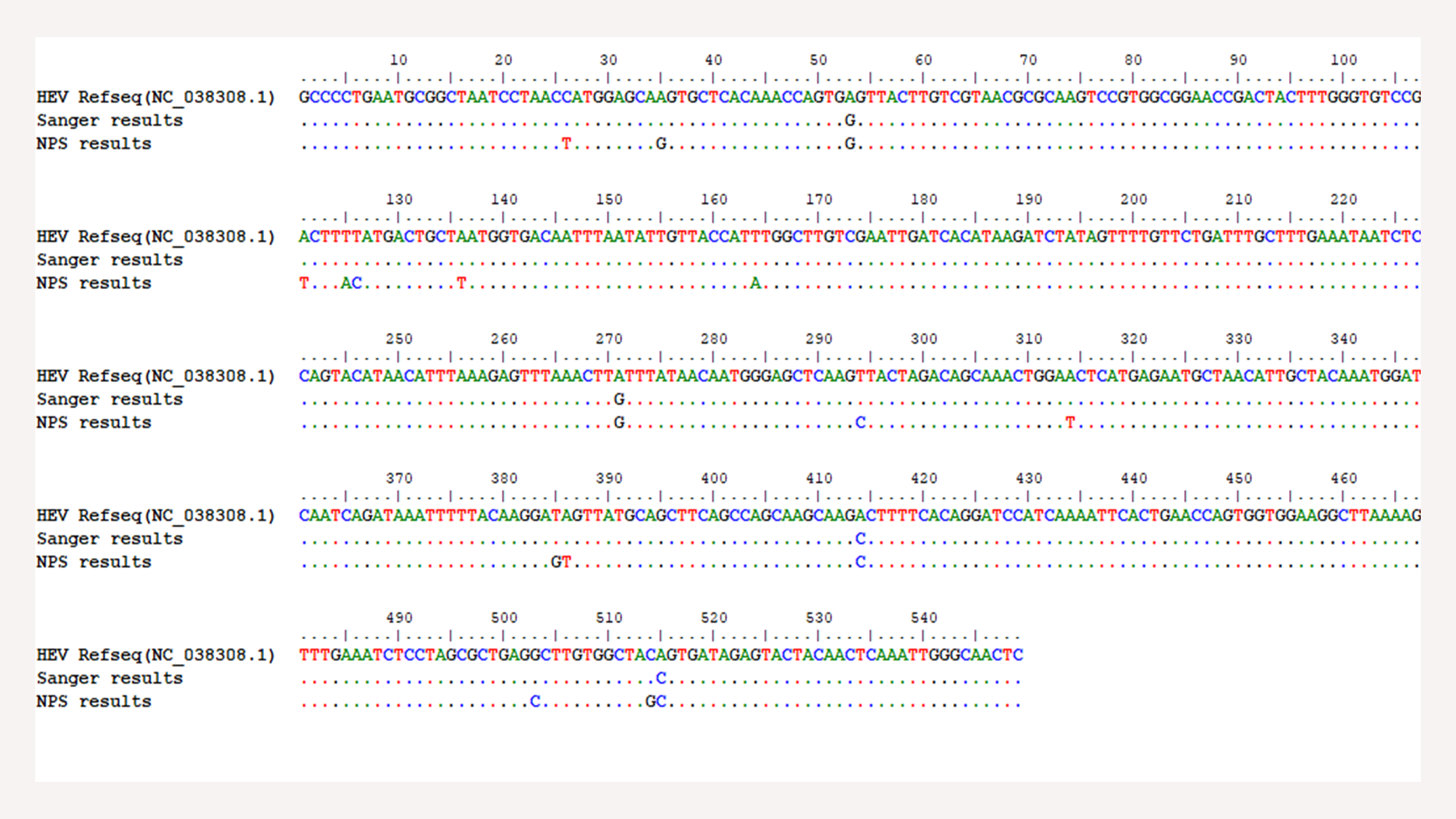

Supplement: S8 Fig — (TIF) [file pone.0324601.s008.tif]

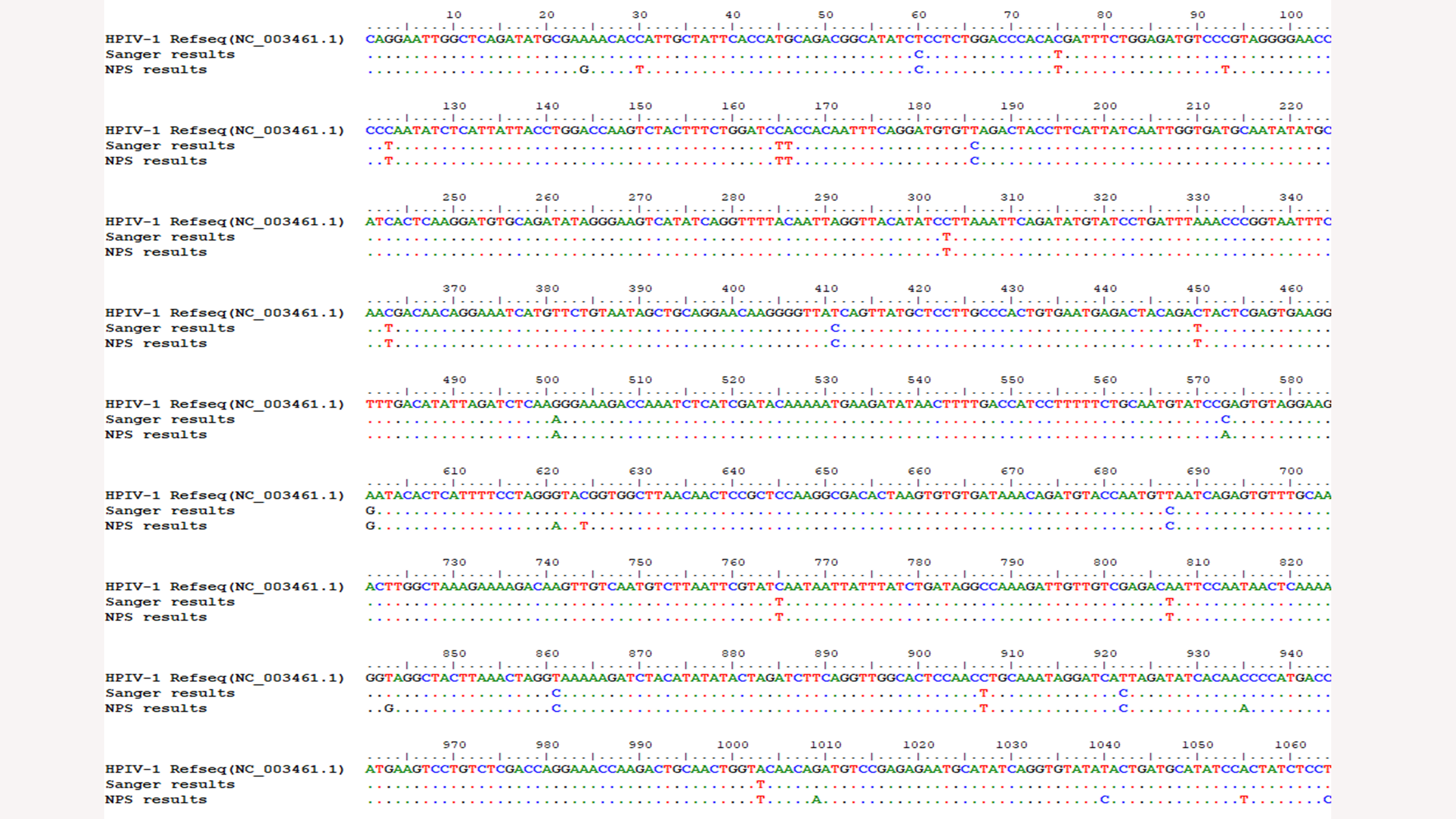

Supplement: S9 Fig — (TIFF) [file pone.0324601.s009.tiff]

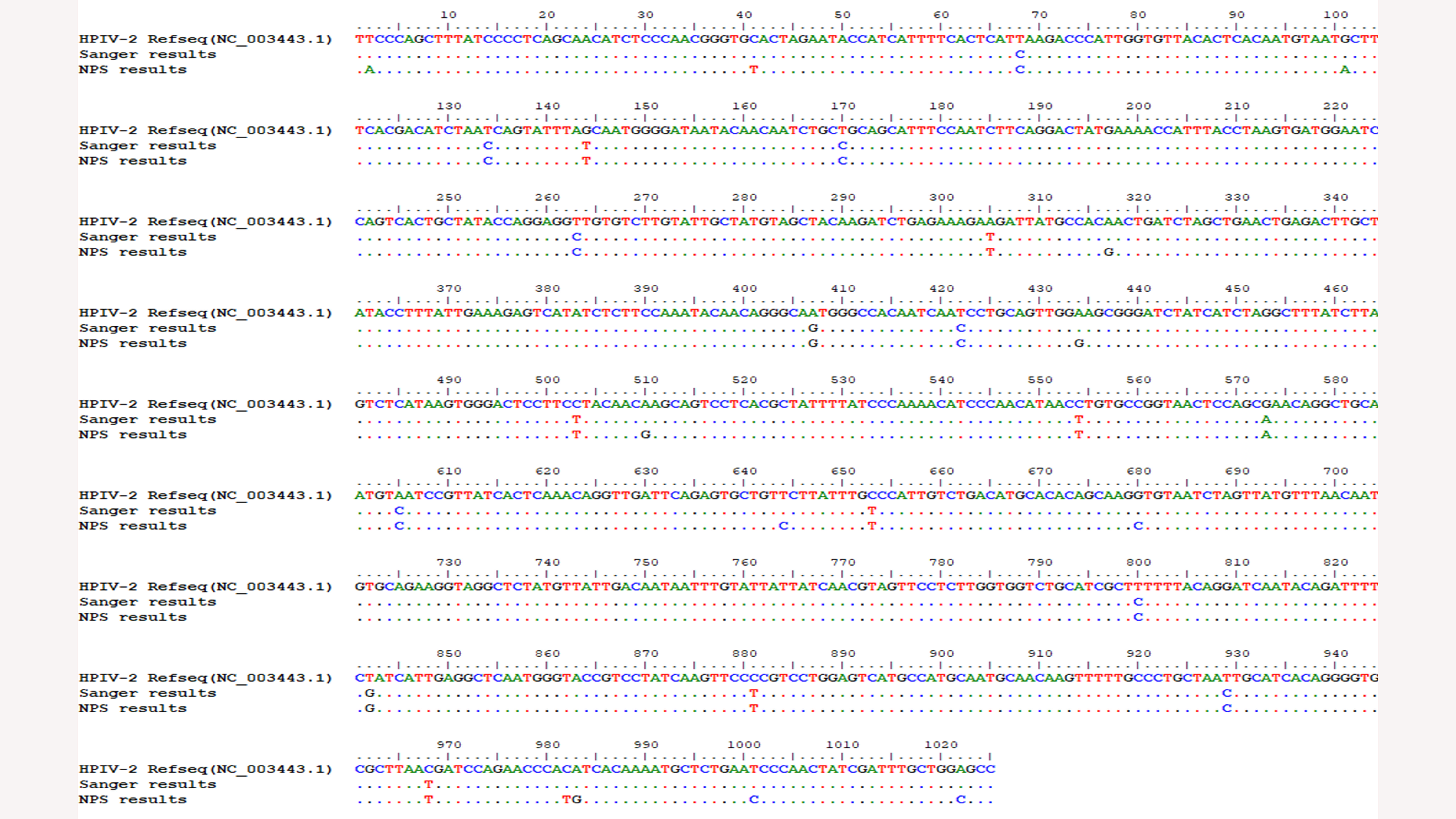

Supplement: S10 Fig — (TIFF) [file pone.0324601.s010.tiff]

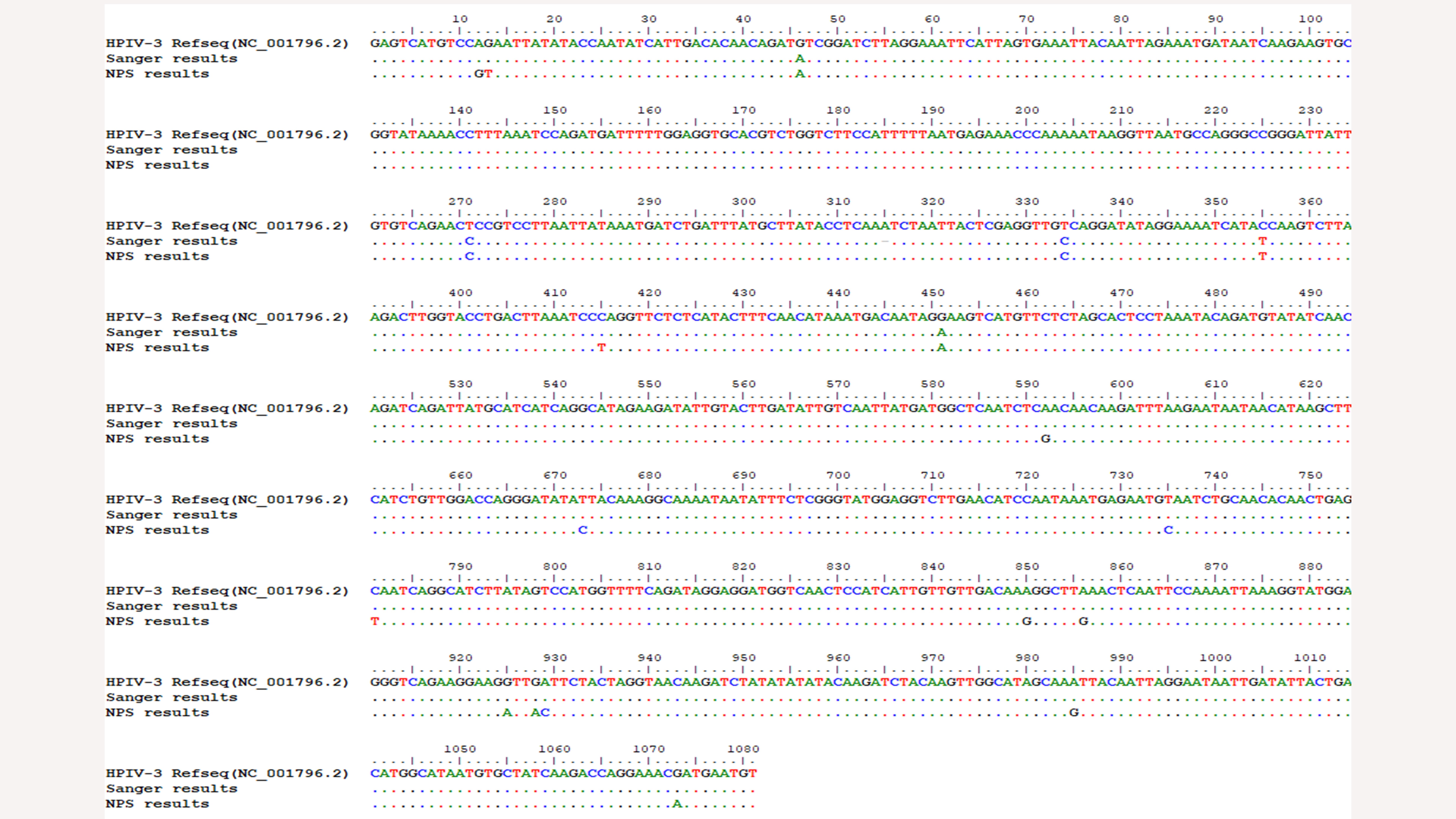

Supplement: S11 Fig — (TIF) [file pone.0324601.s011.tif]
